# WGCNA-based identification of potential targets and pathways in response to treatment in locally advanced breast cancer patients

**DOI:** 10.1515/med-2023-0651

**Published:** 2023-03-06

**Authors:** Ruipeng Zhao, Wan Wei, Linlin Zhen

**Affiliations:** Department of Thyroid and Breast Surgery, The Affiliated Huaian No. 1 People’s Hospital of Nanjing Medical University, Huaian, Jiangsu, China

**Keywords:** WGCNA, breast cancer, immune cell infiltrations, GSEA

## Abstract

Locally advanced breast cancer patients have a poor prognosis; however, the relationship between potential targets and the response to treatment is still unclear. The gene expression profiles of breast cancer patients with stages from IIB to IIIC were downloaded from The Cancer Genome Atlas. We applied weighted gene co-expression network analysis and differentially expressed gene analysis to identify the primary genes involved in treatment response. The disease-free survival between low- and high-expression groups was analyzed using Kaplan–Meier analysis. Gene set enrichment analysis was applied to identify hub genes-related pathways. Additionally, the CIBERSORT algorithm was employed to evaluate the correlation between the hub gene expression and immune cell types. A total of 16 genes were identified to be related to radiotherapy response, and low expression of SVOPL, EDAR, GSTA1, and ABCA13 was associated with poor overall survival and progression-free survival in breast cancer cases. Correlation analysis revealed that the four genes negatively related to some specific immune cell types. The four genes were downregulated in H group compared with the L group. Four hub genes associated with the immune cell infiltration of breast cancer were identified; these genes might be used as a promising biomarker to test the treatment in breast cancer patients.

## 1 Introduction

Breast cancer is the most prevalent malignant tumor among women and is the primary cause of most cancer-related deaths in women [1,2]. In recent years, breast cancer survival rates and prognosis have improved due to novel therapeutic options, new surgical techniques, and a better understanding of this disease [3,4]. Poor molecular typing, such as HR receptor-negative, HER-2 positive, or locally advanced breast cancer, is the main factor affecting the prognosis of breast cancer patients [5]. Currently, radiotherapy and chemotherapy are commonly used for locally advanced patients. Radiotherapy is the standard treatment in patients with breast cancer with positive axillary lymph nodes [6]. Although chemoradiotherapy has some advantages in treating breast cancer, there will still be heterogeneity in the efficacy of radiation therapy, which seriously influences the quality of life and clinical efficacy of patients [7,8]. Besides, the drug resistance of chemoradiotherapy remains the major cause of the failure of cancer treatment [9]. Thus, investigating the potential targets to evaluate chemoradiotherapy response in breast cancer will be important for personalized therapeutic methods.

Chemoradiotherapy not only destructs cancer cells but also activates the immune system. It has been demonstrated that chemoradiotherapy could exhibit an immunostimulating effect via decreasing the accumulation of infiltrating regulation of T cells, increasing the level of tumor-associated M1 macrophages, and increasing NK cell cytotoxicity [10,11]. Previous reports also have demonstrated that radioresistance decreased the efficacy of radiotherapy via modulation of the tumor microenvironment [12,13]. However, the potential biomarkers and molecular mechanisms of drug resistance in breast cancer are still unclear.

In recent years, high throughput RNA-SEQ technologies have been widely applied in disease mechanism research [14]. Weighted gene co-expression network analysis (WGCNA) is a systematic method to describe the correlation patterns among genes via calculating gene connectivity [15]. WGCNA also could form modules and cluster genes by assessing the relationship between hub modules and external clinical features [15]. Therefore, it is often applied to identify specific modules and potential biomarkers [16,17]. In the present study, we integrated WGCNA and differentially expressed gene (DEG) analyses to identify potential prognostic biomarkers of patients with breast cancer undergoing chemoradiotherapy. Besides, the relationship between infiltrating immune cells and prognostic biomarkers was assessed. This research will provide a better understanding of prognostic biomarkers for breast cancer and help improve prediction accuracy.

## 2 Methods and materials

### 2.1 Collection of raw data

We downloaded the clinical data and corresponding gene expression profiles of patients with breast cancer from The Cancer Genome Atlas (TCGA) database (https://portal.gdc.cancer.gov/). Breast cancer patients with stages from IIB to IIIC were included in this study; all patients were treated with radiation treatment or therapy, and the patients were categorized into low-risk and high-risk groups based on the response of their primary tumor to radiation treatment or therapy. The patients with cancer recurrence after radiation treatment or therapy within 5 years were defined as a high-risk group (H), whereas those Disease-free survival (DFS) time over 5 years were defined as a low-risk group (L). The propensity score matching (PSM) was performed using SPSS 25.0 software (IBM, USA). In this study, age, race, T and N stages, estrogen receptor and progesterone receptor status were set according to clinical data of patients after initial screening, and PSM was performed with a matching tolerance of 0.01 to reduce the statistical bias of high- and low-risk groups caused by differences in enrollment conditions.

### 2.2 Construction of WGCNA network

The gene co-expression network was constructed using the WGCNA R package [15]. The “pickSoftThreshold” function of the WGCNA package was used to assess the value of the powers. A topological overlap matrix (TOM) was generated by transforming the adjacency matrix. The co-expressed gene modules were identified using the dynamic tree cut, and a hierarchical clustering method based on the dissimilarity of the TOM was utilized to visualize the cluster dendrogram of genes. Mode membership and gene significance were calculated for each module. The major functional modules with the highest correlation genes were extracted.

### 2.3 Identification of DEGs

The batch effects were removed by using the “sva” R package. The “limma” package was employed to perform DEG screening. ∣log FC∣ ≥ 0.5 and *p* < 0.05 were used as the screening parameters to identify the DEGs. The distribution of each gene was visualized by a volcano plot and generated using the ggplot2 package of R software.

### 2.4 Identification of hub genes and survival analysis

The overlapping genes between co-expression genes and DEGs were obtained by a Venn tool. The “Survival” R package was applied to carry out univariate and multivariate Cox regression analyses in breast cancer cases to assess the prognostic value of overlapping genes. *p*-value <0.05 was considered significant. We used the “timeROC” R package to draw a time-dependent ROC curve to assess the predictive value of the prognostic signature. The “survminer” R package was applied to further analyze the survival probability of low- and high-expression groups.

### 2.5 Gene set enrichment analysis (GSEA)

We used GSEA 4.0.3 software to perform the GSEA for the hub genes. First, the mRNA expression profiles were divided into two groups (low- and high-expression levels of hub genes) based on the median expression level of hub genes. Then, the C2 KEGG gene sets (c2.cp.KEGG.v7.4.symbols.gmt) of the Molecular Signature Database were applied to perform the enriched functions and pathway analysis in the low- and high-expression groups. Pathways with *p* <0.05 and a false discovery rate <0.05 were considered to be significantly enriched.

### 2.6 Assessment of immune cell infiltration

The gene expression profiles were uploaded to the CIBERSORT website. Then, the 22 types of immune cell infiltration matrix were obtained based on *p* < 0.05. The “ggplot2” package of R software was used to visualize the differences in immune cell infiltration between L and H groups. The correlation of 22 kinds of immune cells was analyzed by using the “complot” package of R software.

### 2.7 Correlation analysis between infiltrating immune cell and hub biomarkers

The Spearman correlation between infiltrating immune cell and diagnostic biomarkers was analyzed using the “ggstatsplot” package of R software, and the results were visualized by the “ggplot2” package.

### 2.8 Immunohistochemistry

We collected 10 patients with locally advanced breast cancer who had relapsed within 5 years and five patients who had not relapsed over 5 years from The Affiliated Huaian No. 1 People’s Hospital of Nanjing Medical University to further verify the results of key gene expression. This study was conducted in accordance with the Declaration of Helsinki (as revised in 2013) and was approved by the Ethics Committee of The Affiliated Huaian No. 1 People’s Hospital of Nanjing Medical University. Immunohistochemistry was performed as described based on a previous study [18]. The primary antibodies (rabbit anti-SVOPL, anti-EDAR, anti-GSTA1, and anti-ABCA13) were purchased from Abcam (Cambridge, UK).


**Ethical statement:** The authors are accountable for all aspects of the work in ensuring that questions related to the accuracy or integrity of any part of the work are appropriately investigated and resolved. This study was conducted in accordance with the Declaration of Helsinki (as revised in 2013) and was approved by the Ethics Committee of The Affiliated Huaian No. 1 People’s Hospital of Nanjing Medical University.

## 3 Results

### 3.1 Clinical characteristics of patients

As shown in Table A1, there were no significant differences in age, AJCC pathologic stage, AJCC pathologic T, AJCC pathologic M, Race, ER, and PR receptor status between the H and L groups (*p* > 0.05). There was a significant difference in Person neoplasm cancer status between the H and L groups (*p* < 0.001).

### 3.2 WGCNA identified potential genes associated with chemoradiotherapy resistance in breast cancer patients

**Figure 1:**
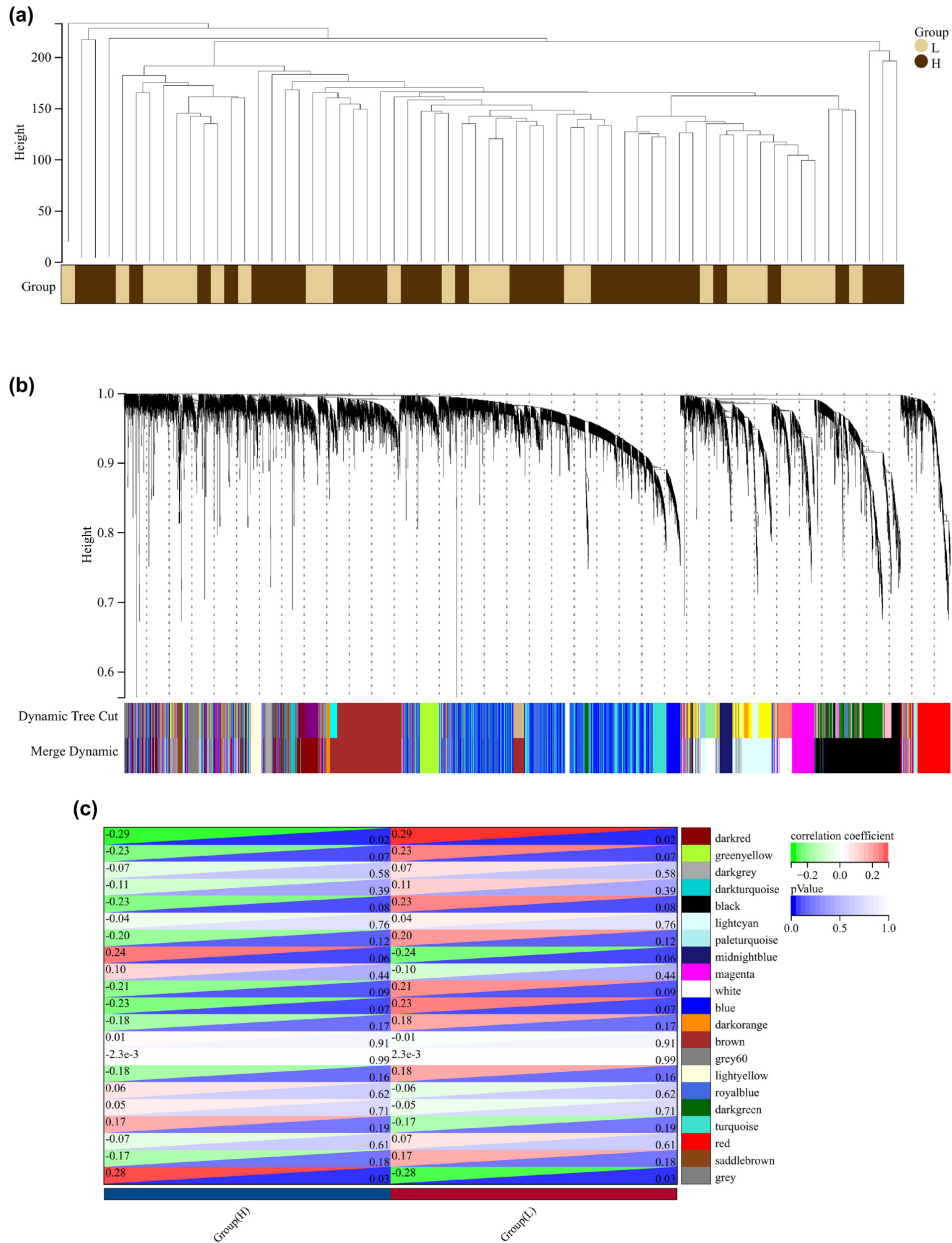
Construction of co-expression modules. (a) Clustering dendrogram of 62 samples. (b) In the cluster dendrogram of genes in the TCGA database, all genes were clustered in 21 modules. (c) Module–trait relationship of two traits and 21 modules.

A total of 16,079 genes collected from 62 samples of TCGA were applied to construct a dendrogram (Figure 1a). We identified 21 modules based on average dynamic tree clipping and hierarchical clustering (Figure 1b). These modules were visualized in Figure 1c, the dark-red (*r* = 0.38, *p* = 4.2 × 10^−13^) and grey (*r* = 0.34, *p* = 1.8 × 10^−3^) modules were most correlated with the radiotherapy response of patients and selected for the next investigation.

### 3.3 Identification of hub genes in the TCGA cohort

**Figure 2:**
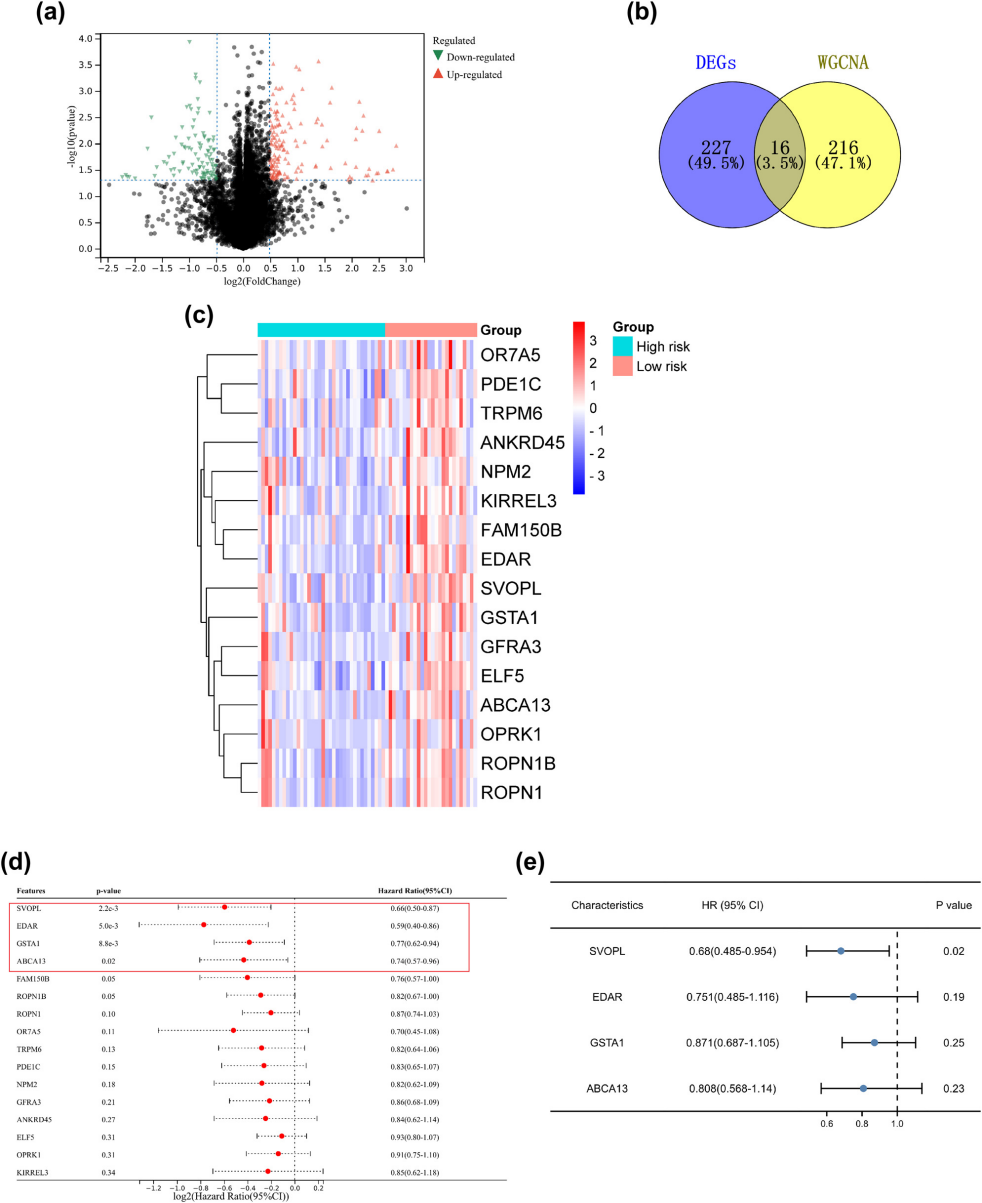
Identification of chemoradiotherapy resistance-related genes in breast cancer. (a) The volcano diagram of DEGs. (b) Identification of hub genes in co-expression network and DEG network. (c) The heat map of 16 hub genes expression between low-risk and high-risk groups. The survival of breast cancer patients was performed by (d) univariate and (e) multivariate cox regression analysis.

A total of 243 DEGs were identified from the gene expression profiles of breast cancer patients, which contains 99 downregulated genes and 144 upregulated genes (Figure 2a). Then, 16 interaction genes were obtained by both DEGs and WGCNA (Figure 2b and c). Moreover, univariate cox analysis indicated that only four of them (SVOPL, EDAR, GSTA1, and ABCA13) were associated with the overall survival (OS) of breast cancer patients (Figure 2d). The multivariate cox analysis indicated that SVOPL was an independent prognostic risk factor for breast cancer patients (Figure 2e).

### 3.4 ROC and Kaplan–Meier analyses of prognostic biomarkers

**Figure 3:**
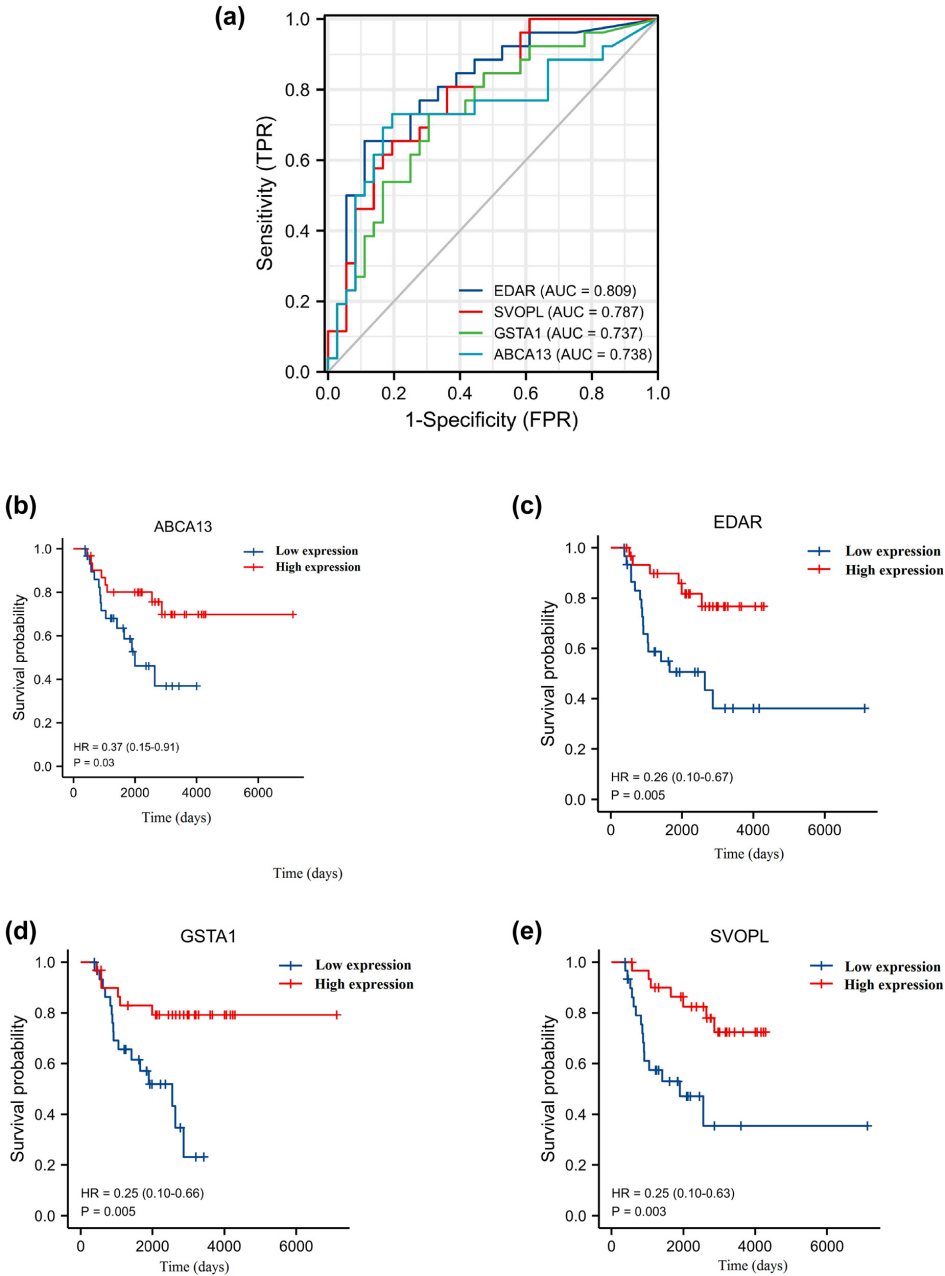
ROC and Kaplan–Meier analyses of prognostic biomarkers of patients with breast cancer undergoing chemoradiotherapy. (a) ROC curves were applied to assess the predictive ability of the hub genes in breast cancer. Kaplan–Meier OS analysis of (b) ABCA13, (c) EDAR, (d) GSTA1, and (e) SVOPL in breast cancer.

As shown in Figure 3a, the ROC curves of SVOPL, EDAR, GSTA1, and ABCA13 showed their probability as valuable genes with AUC of 0.787, 0.809, 0.737, and 0.738, respectively, which indicates the four hub genes had a good predictive value.

**Figure 4:**
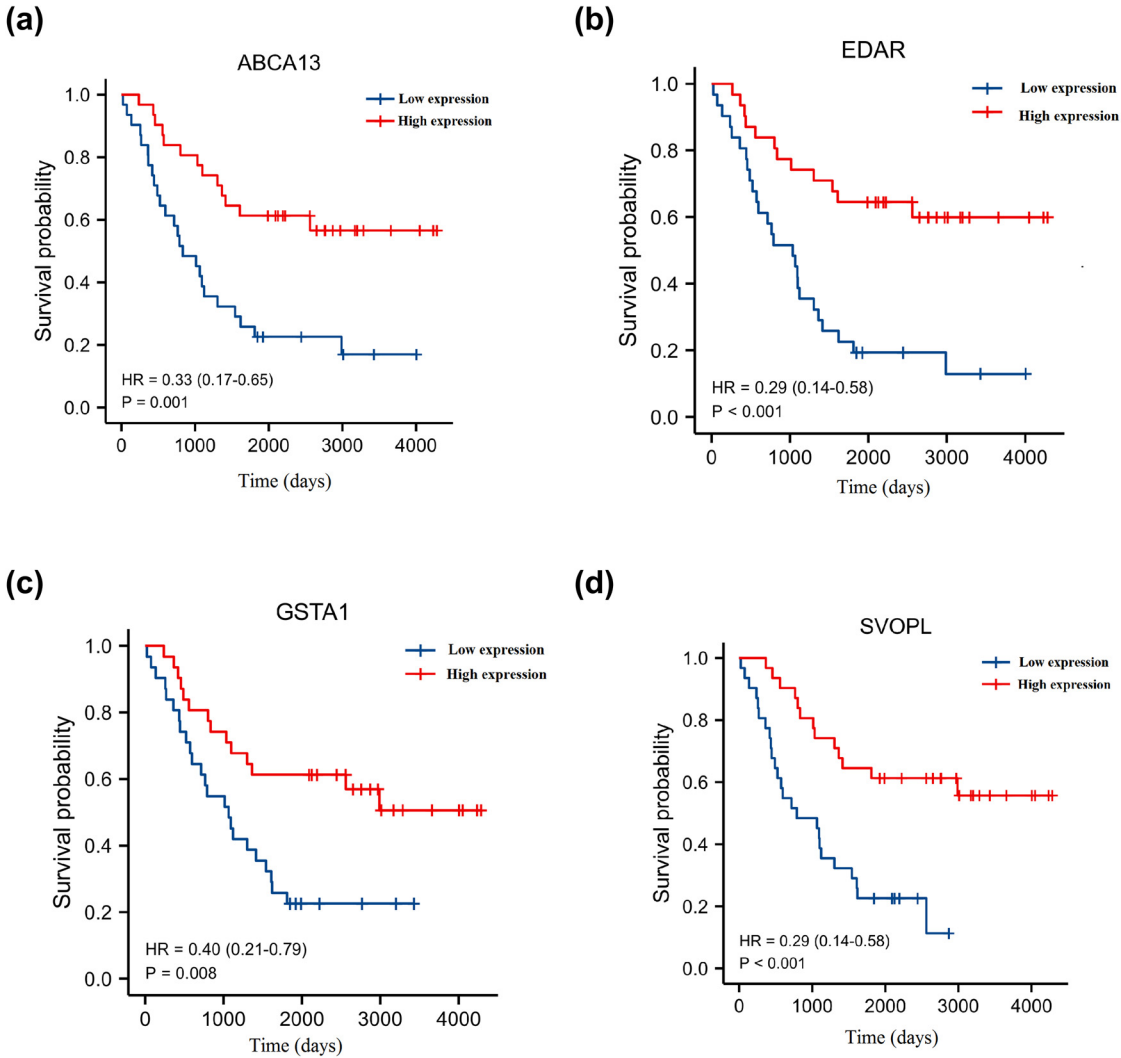
Kaplan–Meier progression-free survival (PFS) analysis of (a) ABCA13, (b) EDAR, (c) GSTA1, and (d) SVOPL in breast cancer.

**Figure A1:**
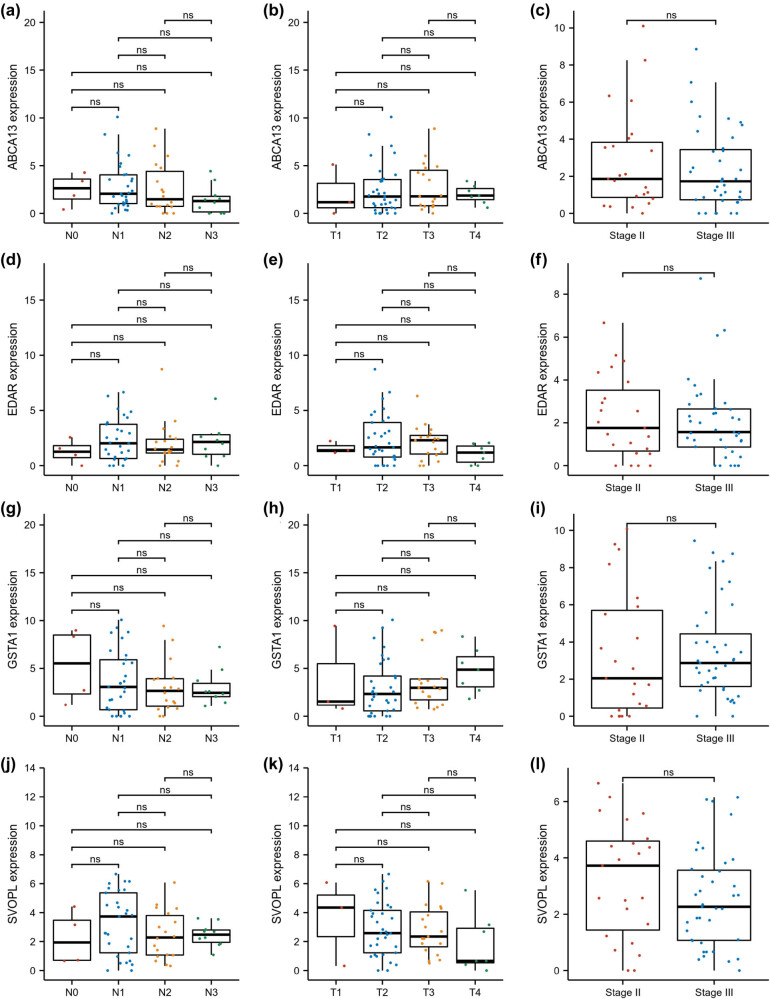
Association between ABCA13, EDAR, GSTA1, and SVOPL expression and tumor stage of breast patients.

Besides, we also investigated the prognostic value of hub genes. As shown in Figure 3b–e, the lower expression of ABCA13, EDAR, GSTA1, and SVOPL were associated with poorer OS (*p* < 0.05). As shown in Figure 4a–d, the lower expression of ABCA13, EDAR, GSTA1, and SVOPL was associated with poorer progression-free survival (PFS) (*p* < 0.05). As shown in Figure A1, the ABCA13, EDAR, GSTA1, and SVOPL expression showed no significant correlation with N stage, T stage, and tumor stage (*p* > 0.05).

### 3.5 GSEA identified hub genes-related pathways

**Figure 5:**
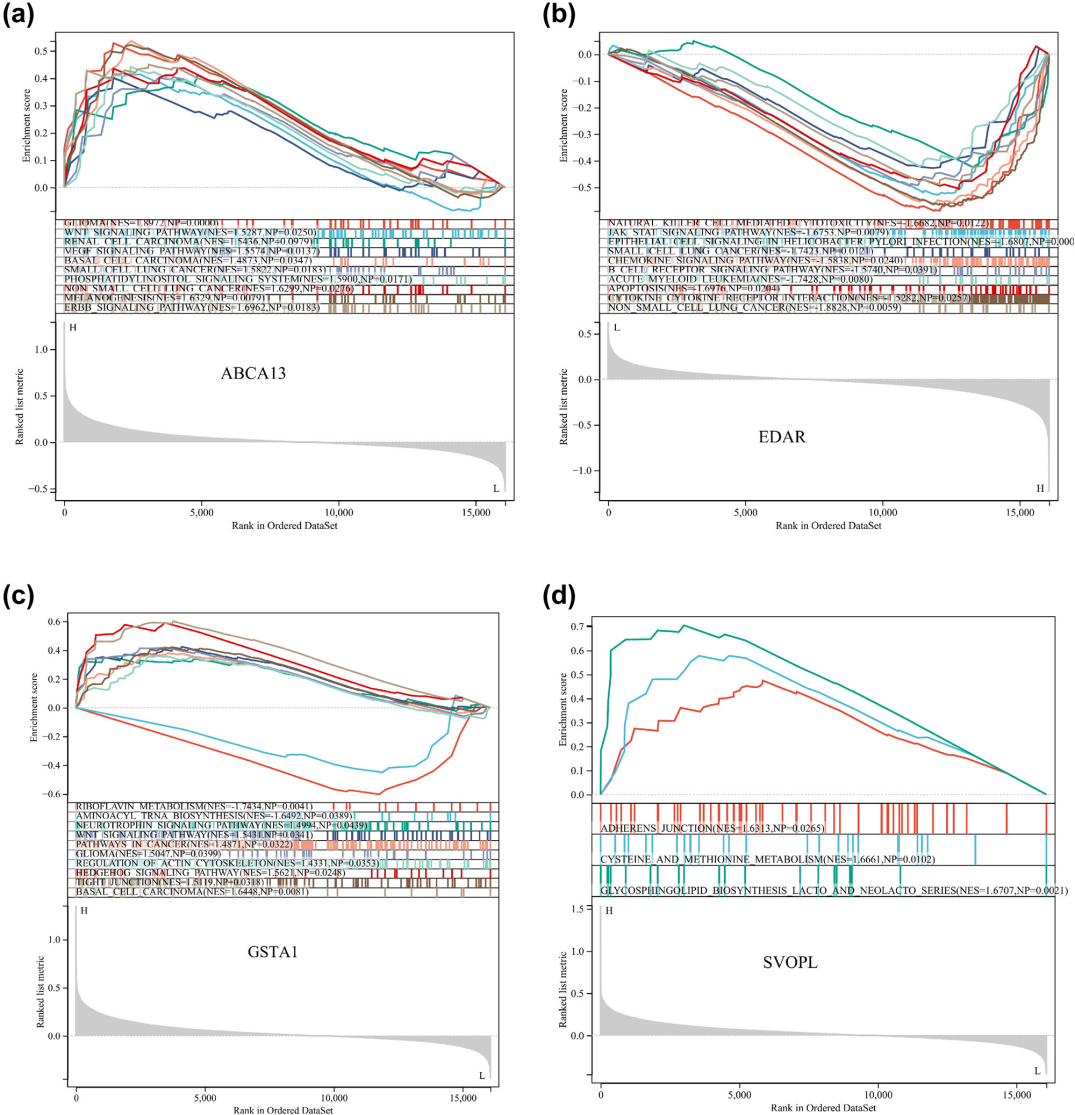
Single-gene GSEA of hub genes. GSEA of (a) ABCA13, (b) EDAR, (c) GSTA1, and (d) SVOPL.

Single-gene GSEA was performed to explore how hub genes are involved in the underlying mechanisms of radiotherapy response in breast cancer patients. As shown in Figure 5a, the glioma, Wnt signaling pathways, renal cell carcinoma, VEGF signaling pathway, basal cell carcinoma, small cell lung cancer, melanogenesis, and ERBB signaling pathway were significantly enriched in the ABCA13 high-expressed phenotype. Natural killer cell-mediated cytotoxicity, JAK-STAT signaling pathway, small cell lung cancer, chemokine signaling pathway, B-cell receptor signaling pathway, acute myeloid leukemia, apoptosis, cytokine, and cytokine receptor interaction, and nonsmall cell lung cancer were significantly enriched in the EDAR high-expressed phenotype (Figure 5b). Wnt signaling pathway, pathways in cancer, glioma, regulation of actin cytoskeleton, tight junction, and basal cell carcinoma were significantly enriched in the GSTA1 high-expressed phenotype (Figure 5c). Adherens junction, cysteine and methionine metabolism, and glycosphingolipid biosynthesis lacto and neolacto series were significantly enriched in the SVOPL high expressed phenotype (Figure 5d).

### 3.6 Analysis of immune infiltrating cells

**Figure 6:**
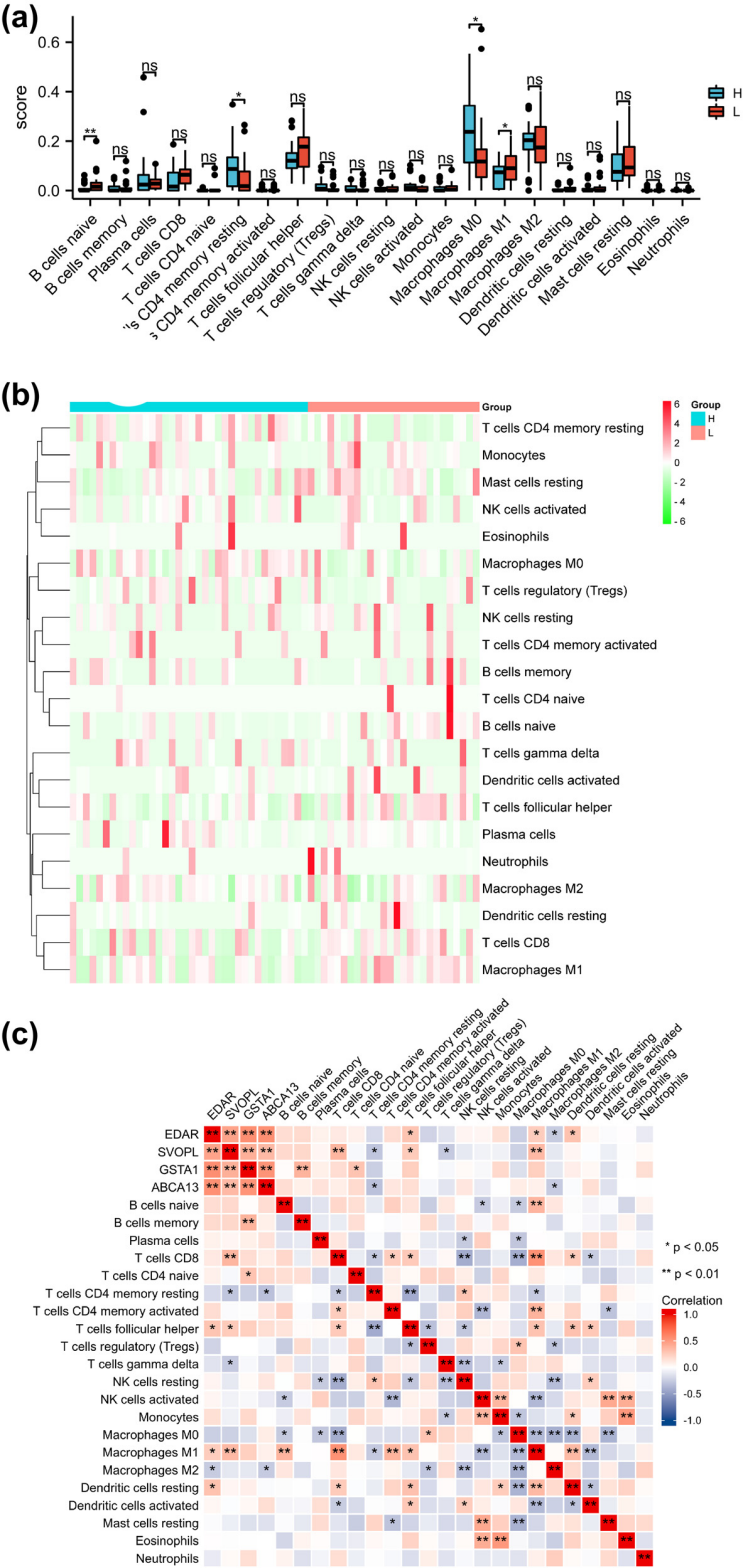
Assessment of immune cell infiltration. (a) Histogram of a score of immune cells between L and H groups. (b) Heatmap of immune cells between L and H groups. (c) The correlation heatmap indicated immune cells. *p < 0.05, **p < 0.01, ***p < 0.001.

As shown in Figure 6a and b, the CIBERSORT algorithm indicated that there were significant differences in naïve B cells, T-cell CD4 memory resting, M0 macrophages, and M1 macrophages between L and H groups (*p* < 0.05). Besides, the correlation heatmap indicated M1 macrophages was positively correlated with naïve B cells, CD8 T cells, and CD4 memory-activated T cells, T follicular helper cells, and dendritic cell resting, whereas M1 macrophages were negatively correlated with T-cell CD4 memory resting, activated NK cells, and M0 macrophages. M0 Macrophages were negatively correlated with naïve B cells, plasma cells, CD8 T cells, monocytes, M1 macrophages, M2 macrophages, dendritic cells resting, and mast cell resting. T-cell CD4 memory resting was negatively correlated with CD8 T cells, T follicular helper cells, and M1 macrophages (Figure 6c).

### 3.7 Correlation analysis between SVOPL, EDAR, GSTA1, ABCA13 expression and tumor immunity in breast cancer

**Figure 7:**
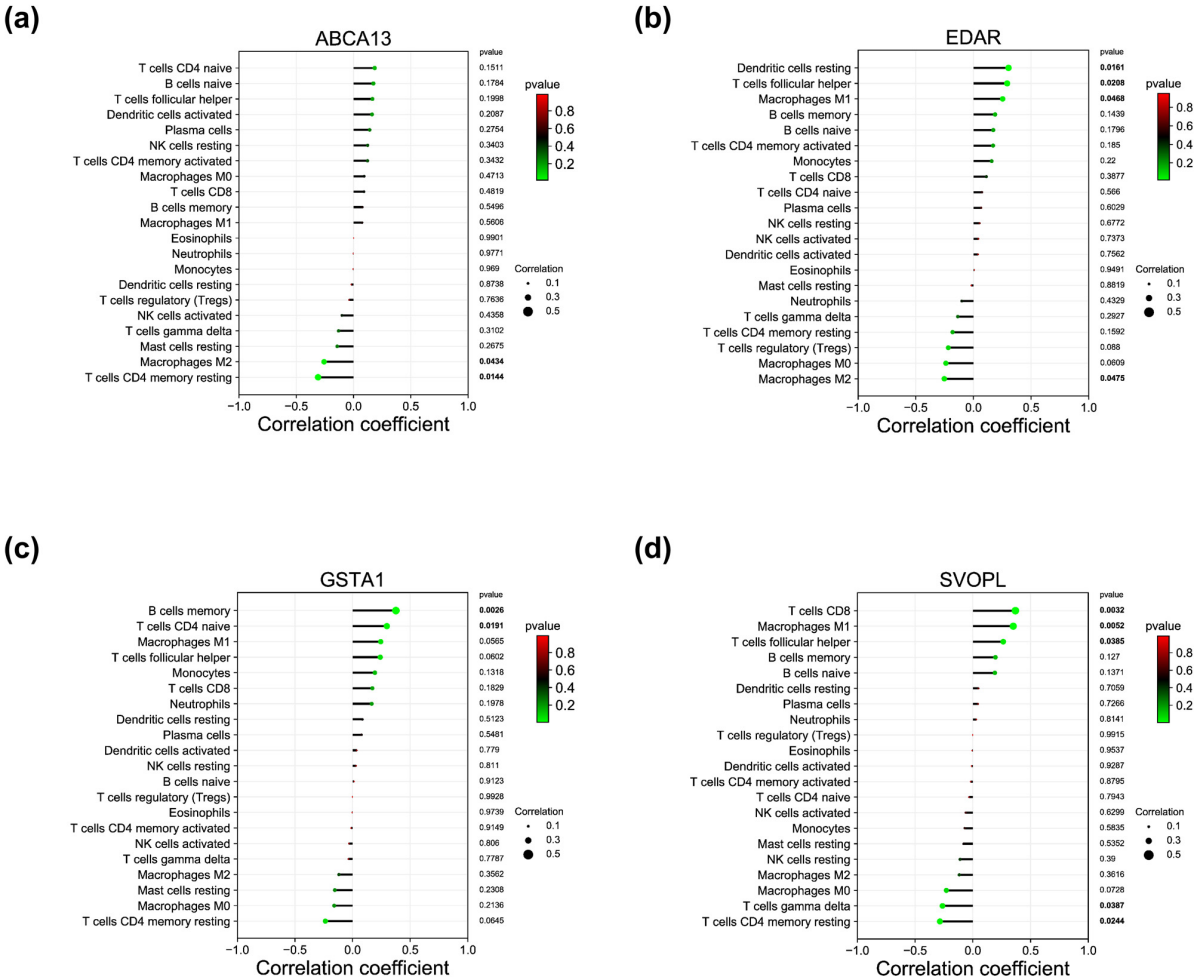
Correlation analysis between infiltrating immune cells and diagnostic biomarkers. The bar diagram showed the correlation between (a) ABCA13, (b) EDAR, (c) GSTA1, (d) SVOPL, and infiltrating immune cells.

According to the results of correlation analysis, ABCA13 exhibited a negative correlation with T-cell CD4 memory resting (*r* = −0.309, *p* = 0.014), and macrophages M2 (*r* = −0.257, *p* = 0.043 (Figure 7a)). EDAR showed a positive correlation with dendritic cells resting (*r* = 0.304, *p* = 0.016), T follicular helper cells (*r* = 0.292, *p* = 0.021), and M1 macrophages (*r* = 0.253, *p* = 0.046) and exhibited a negative correlation with M2 macrophages (*r* = −0.252, *p* = 0.047 (Figure 7b)). GSTA1 displayed a positive correlation with memory B cells (*r* = 0.375, *p* = 0.002), and CD4 naïve T cells (*r* = 0.297, *p* = 0.019 (Figure 7c)). SVOPL showed a positive correlation with CD8 T cells (*r* = 0.368, *p* = 0.003), M1 macrophages (*r* = 0.350, *p* = 0.005), and T follicular helper cells (*r* = 0.263, *p* = 0.038) and showed a negative correlation with gamma delta T cells (*r* = −0.285, *p* = 0.024) and T-cell CD4 memory resting (*r* = −0.263, *p* = 0.038 (Figure 7d)).

**Figure 8:**
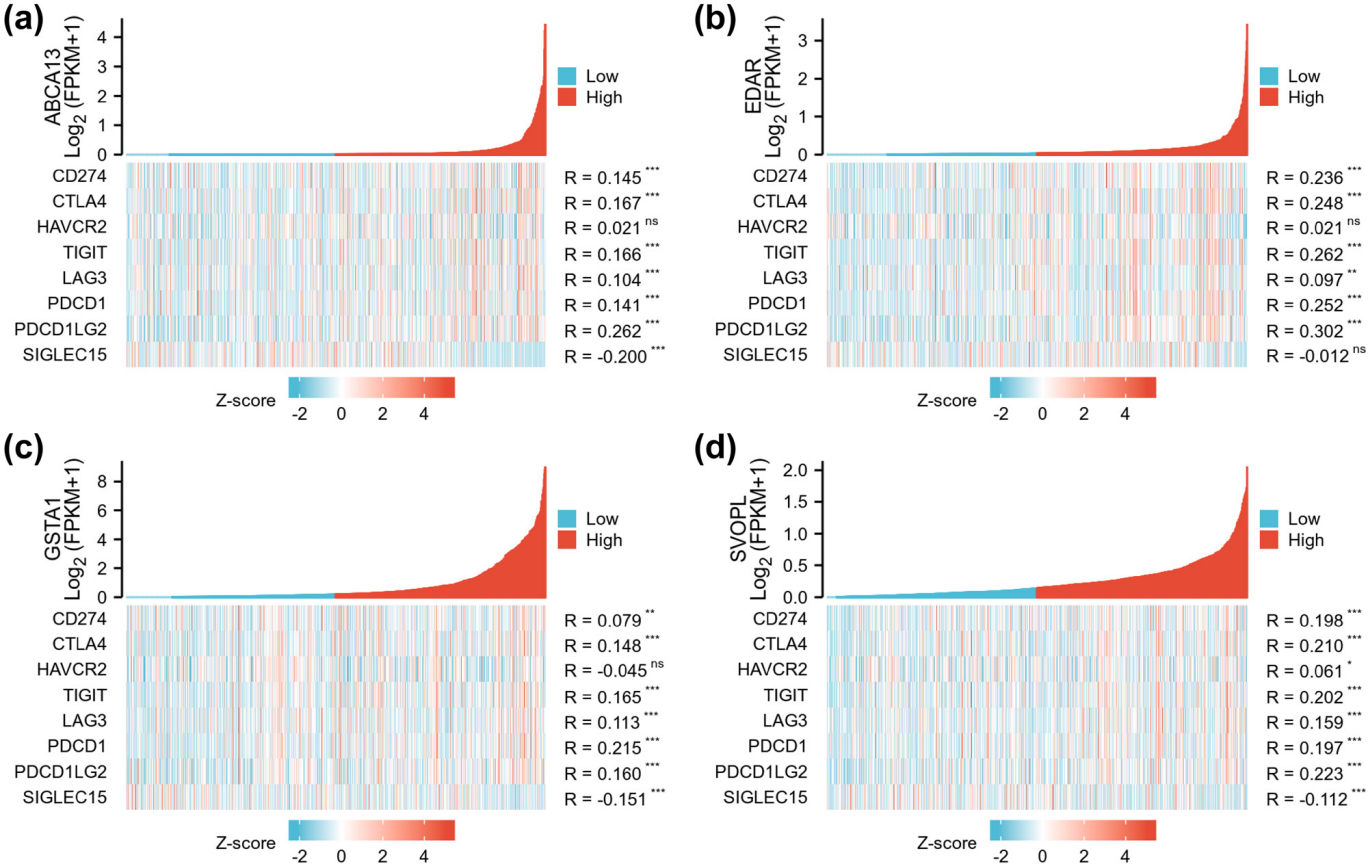
The involvement of ABCA13, EDAR, GSTA1, and SVOPL genes in tumor immunity of breast cancer. The co-expression heatmap presented the correlation between (a) ABCA13, (b) EDAR, (c) GSTA1, and (d) SVOPL expression and eight typical immune checkpoint genes. *p < 0.05, **p < 0.01, and ***p < 0.001.

In addition, the ABCA13, EDAR, GSTA1, and SVOPL expression was positively correlated with six immune checkpoint genes including CD274, CTLA4, TIGIT, LAG3, PDCD1, and PDCD1LG2 (*p* < 0.05, Figure 8a–d). These findings implied that ABCA13, EDAR, GSTA1, and SVOPL may be involved in tumor immunity.

### 3.8 Validation of SVOPL, EDAR, GSTA1, and ABCA13 expression by immunohistochemistry

**Figure 9:**
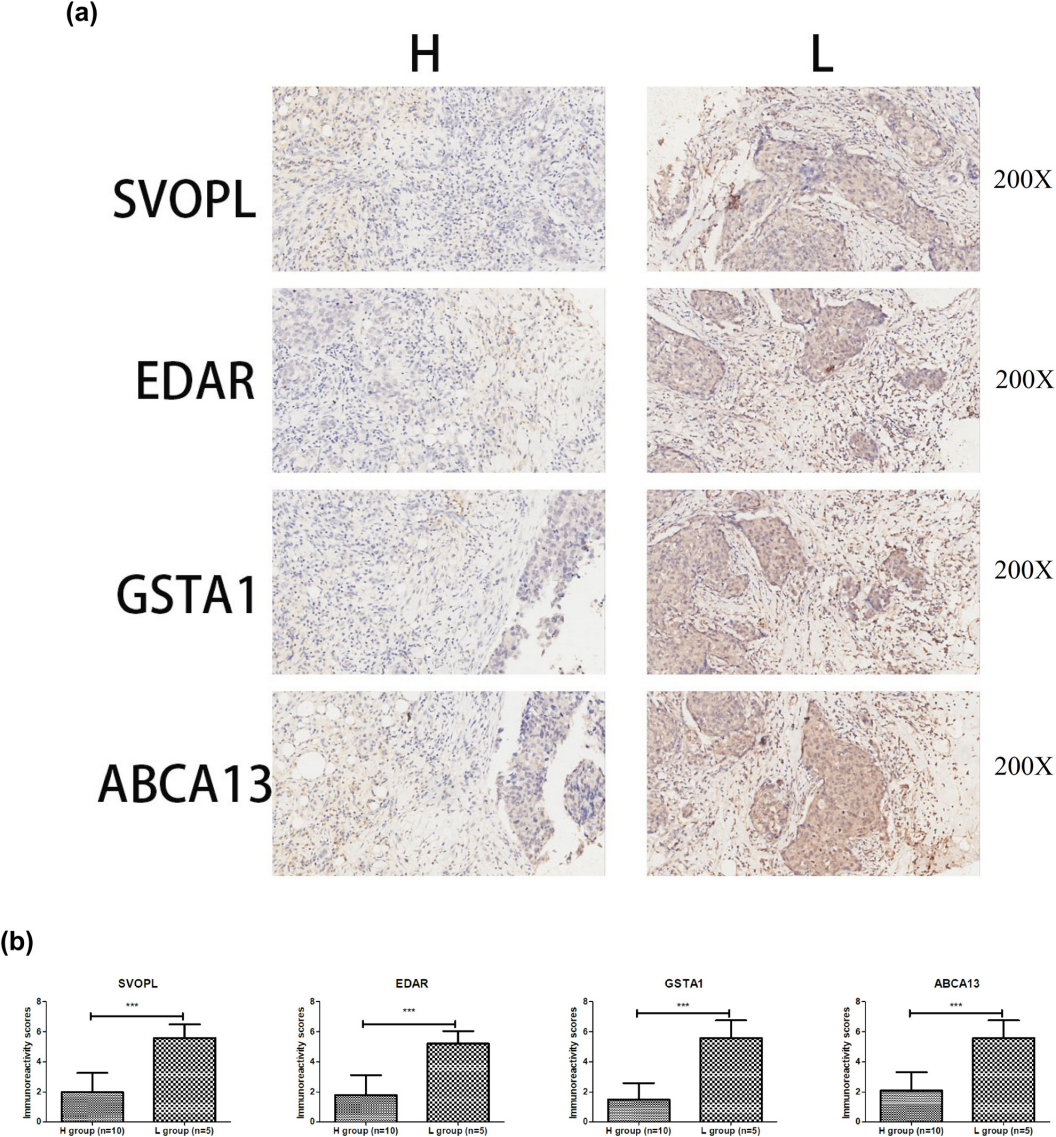
Representative immunohistochemistry images of ABCA13, EDAR, GSTA1, and SVOPL in H and L groups (a). The immunoreacivity scores of ABCA13, EDAR, GSTA1, and SVOPL in H and L groups (b). ***p < 0.001.

To further confirm the above results, immunohistochemistry was performed to detect the SVOPL, EDAR, GSTA1, and ABCA13 levels in H and L groups. As shown in Figure 9a and b, compared to the H group, the SVOPL, EDAR, GSTA1, and ABCA13 levels was higher in the L group (*p* < 0.001).

## 4 Discussion

Chemoradiotherapy is one of the mainstream therapies in tumor treatment, which has a good therapeutic effect on many kinds of cancer [19,20]. However, resistance to chemoradiotherapy is the main obstacle in the treatment of tumors [21,22]. Besides, ionizing radiation also causes the generation of oxygen radicals and DNA damage repair [23]. Previous reports have revealed that subsequent immune responses could greatly influence the efficacy of chemoradiotherapy [24,25]. Chemoradiotherapy also reconfigures the immunological tumor microenvironment that impacts the differential radiosensitivity of malignant cells [26,27]. Although high throughput RNA–SEQ technologies have been used in the research of breast cancer, the underlying mechanism by which chemoradiotherapy influences the tumor immune microenvironment is still undefined. Besides, the molecular mechanism associated with the chemoradiotherapy response in breast cancer cases is still unclear. There is also a lack of reliable and effective biomarkers to predict the prognosis of breast cancer cases.

In the present study, we identified 16 differentially co-expressed genes via DEG and WGCNA analyses. Subsequently, Cox regression analysis identified four prognosis-related biomarkers, including SVOPL, EDAR, GSTA1, and ABCA13. Some of these are reported as being dysregulated in cancer, including breast cancer. For example, EDAR is a death receptor and plays an important role in the development of teeth, cutaneous glands, and hair follicle [28]. Activated ectodysplasin A receptor (EDAR) signaling pathway causes mammary gland tumorigenesis in mice [29]. Decreased glutathione S-transferase A1 (GSTA1) expression is associated with increased breast cancer mainly among them current smokers and lower consumption of vegetables [30]. It has been reported that functional variation of GSTA1 is involved in the development of radiation-induced fibrosis in patients with breast cancer [31]. ATP-binding cassette protein A13 (ABCA13) contributes to the risk of neurological disorders and showed to be a potential regulator of progression and response to the chemotherapy of mammary gland cancer [32]. The previous report has indicated allelic switching of SVOPL during colorectal cancer tumorigenesis [33].

Immune cell infiltration plays an important role in tumor control, and radiotherapy has been indicated to have a great impact on immune responses [34]. Tumor cells can impact immune cell infiltration after radiotherapy, resulting in radioresistant [35]. Therefore, the CIBERSORT was applied to assess the score of 22 kinds of immune cells between the L and H groups. The infiltration of naive B cells and M1 macrophages decreased, while the infiltration of T-cell CD4 memory resting and M0 macrophages increased, probably revealing associations with the occurrence and progress of chemoradiotherapy resistance. We also found that ABCA13 was negatively correlated with T-cell CD4 memory resting and M2 macrophages. EDAR showed a positive correlation to dendritic cell resting, T follicular helper cells, and M1 macrophages and a negative correlation with M2 macrophages. GSTA1 displayed a positive correlation with memory B cells and CD4 naïve T cells. Previous studies have revealed that activation of B cells contributes to the anti-tumor response in a mouse model of breast cancer [36]. Tumor-infiltrating B cells induce the generation of humoral immune response and help to produce anti-tumor immunity in breast cancer [37]. Inhibition of naive CD4 T cell recruitment into cancer cells might be a promising strategy in breast cancer [38]. Tumor-associated macrophages play a vital role in drug resistance, growth, and progression of breast tumors [39,40]. However, their findings require further experimental evidence to prove the complex interactions between immune cell infiltrations and biomarkers in breast cancer.

In conclusion, we identified that SVOPL, EDAR, GSTA1, and ABCA13 are potential prognostic biomarkers of patients with breast cancer undergoing chemoradiotherapy. Our results also indicated that chemoradiotherapy resistance of breast cancer may be associated with the tumor immune cell infiltration, especially M1 macrophages, T-cell CD4 memory resting, and M2 macrophages.

## Appendix

**Table A1:**

Characteristic	High risk group	Low risk group	p
*n*	36	26	
ER, *n* (%)			0.432
Negative	15 (25%)	8 (13.3%)	
Positive	19 (31.7%)	18 (30%)	
PR, *n* (%)			0.394
Negative	18 (30%)	10 (16.7%)	
Positive	16 (26.7%)	16 (26.7%)	
Person neoplasm cancer status, *n* (%)			**<0.001**
TUMOR FREE	5 (8.3%)	25 (41.7%)	
WITH TUMOR	29 (48.3%)	1 (1.7%)	
AJCC pathologic stage, *n* (%)			0.197
Stage IIB	11 (17.7%)	12 (19.4%)	
Stage III	2 (3.2%)	0 (0%)	
Stage IIIA	11 (17.7%)	11 (17.7%)	
Stage IIIB	5 (8.1%)	1 (1.6%)	
Stage IIIC	7 (11.3%)	2 (3.2%)	
AJCC pathologic T, *n* (%)			0.705
T1	1 (1.6%)	1 (1.6%)	
T1c	1 (1.6%)	0 (0%)	
T2	17 (27.4%)	16 (25.8%)	
T3	11 (17.7%)	8 (12.9%)	
T4	1 (1.6%)	0 (0%)	
T4b	4 (6.5%)	1 (1.6%)	
T4d	1 (1.6%)	0 (0%)	
AJCC pathologic M, *n* (%)			0.450
M0	30 (48.4%)	24 (38.7%)	
MX	6 (9.7%)	2 (3.2%)	
Race, *n* (%)			0.688
asian	2 (3.3%)	3 (5%)	
black or african american	7 (11.7%)	4 (6.7%)	
white	25 (41.7%)	19 (31.7%)	
